# An Exceptional Adenocarcinoma in a Girl

**DOI:** 10.1155/2018/4017043

**Published:** 2018-04-01

**Authors:** Bangaly Traore, Ibrahima Kalil Cisse, Malick Bah, Ahmed Monzomba Keita

**Affiliations:** ^1^Surgical Oncology Unit, Donka University Hospital, Faculty of Medicine, University of Conakry, Conakry, Guinea; ^2^Laboratory of Anatomo-Pathology, University Hospital Centre of Donka, Faculty of Medicine, University of Conakry, Conakry, Guinea

## Abstract

Anal adenocarcinoma is very rare and usually occurs in the elderly. We present a case of a 12-year-old girl with an anal margin painful tumor infiltrating the lower rectum, with perineal and vulvar permeation nodules and bilateral fixed inguinal and iliac lymph nodes. Histology showed anal adenocarcinoma with mucosecreting component and independent cells. She had no extra pelvic metastasis on CT scan. She underwent a colostomy and palliative care. This exceptional case challenges us on the diversity of forms of anal cancers that require a multidisciplinary approach. The precarious social context and the age of onset make it difficult to manage this rare cancer.

## 1. Background

Anal cancers are rare. They represent 1.5 to 2.5% of digestive tract cancers [1, 2] and 6% of anorectal cancers [2, 3]. Most are squamous cell carcinomas, accounting for less than 10% of all anal cancers [4]. Anal adenocarcinoma is considered more aggressive than squamous cell carcinoma (SCC) [4]. More than two-thirds occur after age 65. Anal adenocarcinoma occurrence in children is very rare. In this study, we discuss one case of anal adenocarcinoma in a 12-year-old girl.

## 2. Case Presentation

A 12-year-old girl, whose parents are separated, was sent to us in May 2017 for painful swelling and pruritus of the anal margin. Eight months before the first signs, a notion of multiple anal intercourses is reported. Because of the notion of anal intercourses, she was examined in several hospital and social departments without successful outcomes.

**Figure 1:**
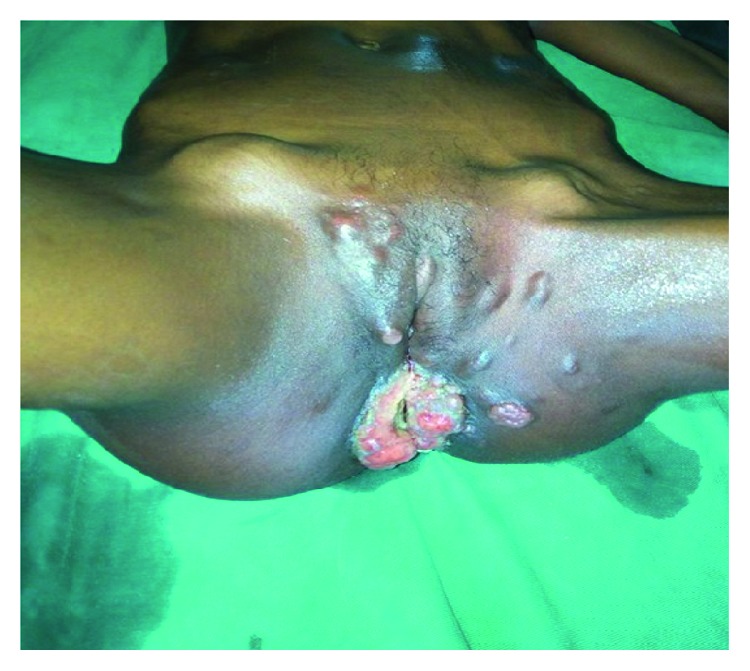
Anal margin tumor with perineal and vulvar permeation nodules and bilateral inguinal lymph nodes.

She presented a large circumferential tumor, budding, ulcerated, and painful tumor of the anal margin, with perineal and vulvar permeation nodules and bilateral fixed inguinal and iliac lymph nodes (Figure 1).

**Figure 2:**
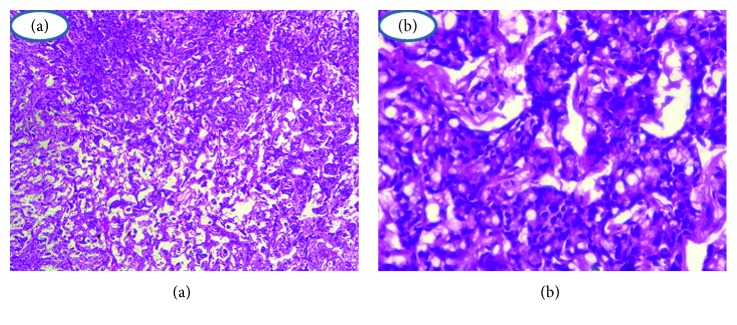
Anal adenocarcinoma with mucosecreting component and independent cells.

**Figure 3:**
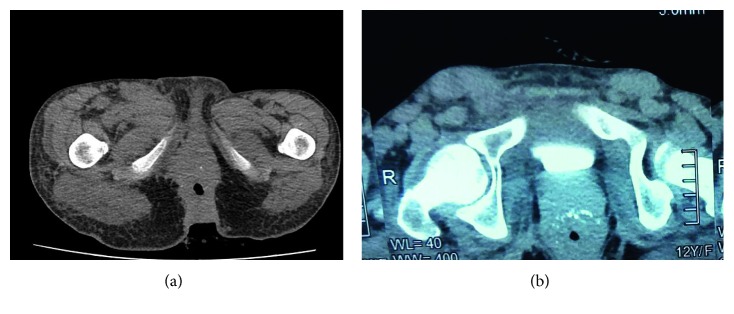
Anal and rectocanal thickening with inguinal and iliac lymph node involvement on CT scan.

Histology showed anal adenocarcinoma with mucosecreting component and independent cells (Figure 2). Immunohistochemical study (IHC) was not performed. The patient was negative for human immunodeficiency virus (HIV). She is anemic. Computed tomography (CT) showed anal and rectocanal thickening with inguinal and iliac lymph node involvement (Figures 3(a) and 3(b)); there was no extra pelvic metastasis.

For our 12-year-old girl who presents an ADK of the anus classified cT4N3M0, we realized a colostomy in gun barrel. Anemia was corrected after transfusion of globular concentrate. Chemotherapy could not be administered because of lack of financial resources. Evolution without anticancer treatment was a locoregional tumor progression. The patient is under tramadol to calm the sometimes intense perineal pain.

## 3. Discussion

Anal adenocarcinoma occurrence in a 12-year-old child is extremely rare. The average age is 65 years or older [5, 6]. To our knowledge, our patient could be the youngest patient worldwide. Anal adenocarcinoma can be separated into three main categories: tumors arising from the mucosal surface, anal glands, or along fistulous tracts [1, 4]. The origin is the anal mucosa and rarely as in our patient, perianal. This patient has no family history of cancer. Anal intercourse could be the risk factor. This notion of sexual intercourse should have the human papillomavirus DNA detected on the tumor tissue whose role is reported by some authors [7]. Other risk factors (tobacco, HIV, Cronh's disease, and chronic fistula) [8, 9] are not found in our patient. The clinical signs are the same as those of SCC of the anus. Mass, anal pain, and pruritus were the signs of onset in our patient. But other signs such as hemorrhage anal discharge may be associated [10]. It is difficult to differentiate anal cancer from the lower rectal cancer with invasion of the anus in this 12-year-old patient. We did not perform immunohistochemistry, but the cytokeratin (CK20) is positive for rectal type and negative for perianal gland type, while CK7 will be positive in perianal type and variably positive in rectal type [8, 11, 12]. But clinical history allows us to retain the anus as a primitive site in our patient. She was seen in stage III of her disease, while more than 61% of Chang et al.'s [10] patients were diagnosed with stages I and II. The advanced stage is due to the precarious social context and several detours in the hospital services without better multidisciplinary approach. Current adenocarcinoma management is controversial, and experience with this disease remains largely anecdotal or based on very small series. The goal standard of anal adenocarcinoma treatment is surgery framed by radiochemotherapy or chemotherapy [4, 5, 10, 13, 14]. Surgical treatment depends on tumor size, stage, and physical performance. Wide excision is reserved for tumors smaller than or equal to 2 cm, and then abdominoperineal amputation is indicated for tumors greater than 2 cm [10]. In our patient who had a large tumor of the anus, infiltrating the perineum, the vulva, the anal canal, and the lower rectum, we performed a colostomy. This palliative surgery made it possible to reduce the anal pain while waiting for the chemotherapy, which was never carried out because of the financial problems. In this context of privileged palliative care, radiotherapy and/or surgical treatment are only discussed according to the outcomes of neoadjuvant chemotherapy. We think that radiotherapy would be poorly indicated because of the growth cartilages in this 12-year-old girl. If chemotherapy was available, we would have administered the FOLFOX4 protocol and, depending on the clinical response, performed a posterior pelvectomy and bilateral inguinoiliac lymph node dissection.

## 4. Conclusion

This exceptional case challenges us on the diversity of forms of anal cancers that require a multidisciplinary approach. The precarious social context and the age of onset make it difficult to manage.
